# The Radcliffe Infirmary, Oxford

**Published:** 1916-07-29

**Authors:** 


### THE DEPARTMENT OF MODERN NURSING.*

### The Radcliffe Infirmary, Oxford.

This is not one of the hospitals which has to go begging for probationers. There is a good choice of candidates and no deterioration in the quality of those applying has been observed. We have often noticed that when this is the case there is accompanying a certain accessibility on the part of the matron towards those interested in the hospital. The Radcliffe Infirmary has not only its own unique standing as the centre of medical research in the oldest English university, it is also a centre for highly organised philanthropic and social endeavour. The formalism and exclusiveness which sometimes repel the public in county hospitals find no place in this aggregation of buildings, old and new, so graciously set in their own grounds that they form an interesting feature among all the architectural treasures of the town. A large circle of supporters make hospital interests their own, and wherever this is the case probationers flow in naturally, for their source, after all, is the public itself.

### The Choice of Candidate.

In former days all probationers paid a premium, but this has been allowed to drop into abeyance, for here, as elsewhere, the limitation of choice to women able to afford a premium has not been found to the advantage of the institution. In a university town it would be natural to expect that some women who had completed their academic course might be disposed to take up hospital training, and this has been the case, though not to any large extent. The term of training is for three years, at a salary of £5, £10, and £20 per year respectively. Pupils can be received for one year's training on payment of £40.

### The Probationers' Course.

After two months' trial in two different wards the probationer begins her regular course of training. The ward scheme is as follows: During the first year probationers are on day duty two months at a time, so that they get six changes of ward. They may, if required, have short spells of night duty during this time, but, as a rule, during their first year they have very little night duty. During the second year they are three months at a time on day and night duty alternatively. In the third year they are staff nurses, and remain, as a rule, in the same ward for six months, being three months on day and three months on night duty in it. Naturally, this scheme is subject to modifications.

### Lectures and Examinations.

Hitherto the probationers have not had their own lecture-room, but among the recent additions this very desirable one has found a place, and the work of the training school is greatly facilitated in consequence. Fifteen lectures on elementary physiology and nursing are given by the clinical physician, and fifteen lectures on elementary anatomy and surgical nursing by one of the surgeons of the hospital. It is not clear that sufficient emphasis has been laid in the past on the necessity for sound theoretical instruction, and with the better accommodation now available it is hoped to bring this branch of the training into greater prominence. The appointment of an outside examiner would probably have an excellent effect in stimulating the energies of the probationers.

### Practical Teaching.

There are ten sisters, and the system in force brings them into close touch with their probationers, and ensures excellent attention to detail. The practical points with which probationers must become especially familiar are issued in a typed list to each sister, and from this list we give the following extract:—

### Special Instructions for Ward Sisters.

It is requested that the sisters of the various wards in this hospital will hold themselves responsible that their nurses—staff, supernumerary, and probationers—shall be taught and become familiar with all details of nursing, according to the section in which they are employed, the sisters kindly noting that medical, surgical, and gynæcological work should be taught from their respective wards, and that nurses should become especially familiar with all the practical points mentioned below:—

Bedmaking.—Use and drawing of draw-sheets; changing of sheets for helpless patients; use of mackintoshes, cradles, knee-pillows, etc.; use of pillows and rests for heart, abdominal, and pneumonia cases; moving and lifting of helpless patients; the care of backs and prevention of bed-sores.

Beds.—Operation, fracture, rheumatism, etc.; water and air pillows and beds; hot-water bottles—care in filling and placing in beds; system of receiving new patients, and rules which have to be observed as to bathing, weighing, attention to head, etc.; observations to make—temperature, urine specimen, and care of clothes.

Baths.—Bathing in bed. A.M. and P.M. washing; washing of hair; hot-air, continuous, local, and special (medicated) baths; sponging and ice cradling; dry, cold, and hot packs.

Applications.—Hot and cold; fomentations, poultices; evaporating lotions; ice bag, etc.; pneumonia jackets; steam kettles and tent beds.

Enemas.—Various—simple, purgative, medicinal, and feeding by rectum.

Injections.—Hypodermic and per rectum; salines; infusions.

Counter Irritants.—Mustard plaster or leaf; blisters; leeches.

Catheters.—Passing and cleansing of; bladder washings and douches; nasal and œsophageal feeding and stomach washing; aspiration and tapping, preparation for, etc., etc.

### Nurses.

Matron will be grateful if the sisters will pay particular attention to the manners of the nurses

* Previous articles: May 16, 30; June 13; July 11, 25; August 8, 1914; January 16, 30; February 27; March 13; April 3; May 8, 22, 29; October 9, 1915; January 8; February 12, 1916.

in the wards and corridors. That no talking takes place from bed to bed, especially forbidding the discussion of patients and their ailments. That their work shall be done with punctuality and as little noise as possible, with due regard to the patients' feelings. That their manner to patients' visitors and visitors to the wards shall be, at all times, courteous and polite. That they shall be taught to realise when on duty in the hospital that they are representing a public institution, and treat with respect the uniform of their profession.

The training in outdoor nursing and tuberculosis is particularly good. Few hospitals offer more charming conditions for open-air life, and it is easy to see, even by passing through the wards, that the patients are treated as individuals, and are sensible of making part of a harmonious system, where their sufferings and wants are dealt with in a kindly spirit. To make open-air life agreeable for sick people requires exceptional organisation, and there can be no better test of a sound nursing system than the nurse's success in remembering idiosyncrasies and forestalling discomforts.

The massage sister's duties include also the special coaching of probationers in the subjects of their lectures. A course of massage is now available for certain selected nurses. Cooking is taught to both junior and senior probationers by an instructor from the technical school, and their progress is tested by examination. The cooking school is held in the servants' hall in the evening, and is very popular. Each probationer receives, as a rule, at least three months' theatre training during her first two years, and as a staff nurse she gets ample opportunities of perfecting herself in this branch of work.

Each nurse has a separate room, and these are all unusually good and charmingly fitted up. The community rooms are also good, and the reading-room is well furnished with periodicals and papers. There is no lack of interest and recreation in off-duty hours, and the lot of the nurses at the Radcliffe is certainly cast in pleasant places.

### Holidays and Leave.

By the aid of an extra probationer and staff nurse the matron is able to arrange that every probationer has a day off once a fortnight. The system adopted allots the same day of the week regularly to each ward as the off-duty day, so that the nurses in Ward A, for instance, know that every Monday one or other of them will have a day off, her place being filled by the supernumerary. The simplicity and convenience of this system make it worthy of imitation. The time-table for probationers shows an average of nine and a half to ten hours when on day duty, deducting off-duty and meal times, and eleven and a half hours on night duty. Three weeks' holiday are allowed in the year.

### Domestic Economy.

A high level of efficiency has been attained in the housekeeping. According to "Burdett's Hospitals and Charities" for 1916 the average cost of the nurses' board is £17 14s. a year, and the average cost of each nurse to the hospital under the headings of salary, board, and uniforms is £38 14s., a figure which is lower than that of any other hospital of its class. The average cost of the nursing at the Radcliffe per occupied bed is £17, the average number of occupied beds which falls to each member of the day and night staff being 2.64.

